# White matter abnormalities are associated with the declined ability of reasoning and problem‐solving in major depressive disorder

**DOI:** 10.1002/brb3.3047

**Published:** 2023-06-05

**Authors:** Ruihua Ma, Yang Luo, Sijia Liu, Xuan Wang, Hua Guo, Meng Zhao, Nan Chen, Panqi Liu, Jing Shi, Yingna Li, Yunlong Tan, Shuping Tan, Fude Yang, Li Tian, Zhiren Wang

**Affiliations:** ^1^ Peking University HuiLongGuan Clinical Medical School, Beijing Huilongguan Hospital Beijing China; ^2^ Zhumadian Psychiatric Hospital Zhumadian Henan Province China; ^3^ Department of Neurosurgery Sanbo Brain Hospital, Capital Medical University Beijing China; ^4^ Institute of Biomedicine and Translational Medicine, Department of Physiology, Faculty of Medicine University of Tartu Tartu Estonia

**Keywords:** diffusion tensor imaging, major depressive disorder, reasoning and problem‐solving, white matter

## Abstract

**Objectives:**

Executive function in people with depression is linked to the integrity of white matter fibers in the brain. We hypothesized that the maze tests in neuropsychological tests assessed reasoning and problem‐solving abilities dependent on the integrity of brain white matter fibers, and assessed this relationship using diffusion tensor imaging (DTI) in depressed patients and healthy controls.

**Methods:**

Participants aged from 18 to 50 years were recruited from Zhumadian Second People's Hospital from July 2018 to August 2019. The sample included 33 clinically diagnosed individuals with major depressive disorder (MDD) and 24 healthy volunteers (HVs). All subjects underwent Neuropsychological assessment battery (NAB) maze tests and DTI. Tract‐based spatial statistics technology in FSL software was used to process DTI data, and threshold‐free cluster enhancement (TFCE) was used to perform multiple comparison corrections. The fractional anisotropy (FA) of white matter fibers in the MDD group and HVs group were compared and extracted. Pearson correlation was used to analyze the relationship between FA and NAB scores and HAMD scores.

**Results:**

The mean NAB maze test score for the MDD group was lower than the HVs group, and the difference was statistically significant (*F* = 11.265, *p* = .037). The FA value of the body of corpus callosum and cerebral peduncle right in the depression group was lower than that in the healthy control group, and the difference was statistically significant (*p* < .05). FA value of the body of corpus callosum was positively correlated with NAB score (*r* = 0.400, *p* = .036), but not with the HAMD score (*r* = 0.065, *p* = .723).

**Conclusions:**

The decreased ability of reasoning and problem‐solving in MDD may be due to the decreased integrity of the white matter fibers of the body of the corpus callosum.

## Graphical abstract

The NAB maze test score of MDD patients was lower than that of HVs group, The FA value of body of corpus callosum and cerebral peduncle right in depression group was lower than that in healthy control group. FA value of body of corpus callosum was positively correlated with NAB score, but not with HAMD score. The decreased ability of reasoning and problem‐solving in MDD may be due to the decreased integrity of white matter fibers of the body of the corpus callosum.

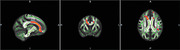

## 1 INTRODUCTION

Depression is the leading cause of disability worldwide, affecting 4.7% of the global population (Daly et al., 2019; Ferrari et al., 2010). Cognitive impairment is a key factor affecting depression. Several studies have shown that around two‐thirds of people with depression experience impaired cognitive functioning (Geraets et al., 2021). As one of the core symptoms of depression, cognitive dysfunction leads to the decline of social function and quality of life of patients with depression, which brings a huge burden to the family (Duric et al., 2018). At present, cognitive impairment has been identified in areas such as executive function, attention, working memory, learning, processing speed, and spatial cognition (Miskowiak et al., 2017; Poletti et al., 2017; Shao et al., 2017). Previous studies have shown that cognitive function, especially executive function, declines significantly during acute episodes of depression (Santos et al., 2014). Executive dysfunction is the most common cognitive deficit in depression (Santos et al., 2014). Research found a negative association between the severity of the major depressive disorder (MDD) and executive function (Lemoult & Gotlib, 2019). In addition, despite the improvement in emotional symptoms, executive dysfunction often persists and takes a long time to recover (Evans et al., 2014). Executive function is a neurocognitive process that continuously and effectively solves a problem to achieve a predetermined goal (Hammar, 2009). Decreased executive function in patients with depression has been frequently reported, and deficits have been shown in many aspects such as working memory, reasoning, and problem‐solving (Price & Duman, 2020). Social problem‐solving reflects the process in which people propose effective solutions to the problems encountered in daily life (Ruan et al., 2022). The ability to solve these problems effectively is important not only for our relationships with others, but also for our mental health and spiritual well‐being (Goddard et al., 1996).The reduced ability to solve social problems is a key characteristic of people with depression (Goddard et al., 1996). Studies have found that people with depression show a negative tendency toward social problems compared to healthy individuals (Noreen & Dritschel, 2022; Priester & Clum, 1993). Rumination may be a key mechanism for poor social problem‐solving in depressed patients, but its biological mechanism remains to be explored (Nolen‐Hoeksema, 1991). Reasoning and problem‐solving are important for people with depression to adapt to society. It is important to explore biomarkers of impaired reasoning and problem‐solving in depression to deepen our understanding of depression.

Studies have shown that abnormal white matter microstructure in schizophrenia is associated with cognitive impairment. Reduced cognitive function in patients with depression is also associated with white matter impairment (Charlton et al., 2014; Yamada et al., 2022). Cognitive decline may be due in part due to damage to white matter caused by damage to blood vessels and glial progenitor cells (Chapman et al., 2016). Recent studies have shown changes in certain structural and functional networks of specific depressive symptom dimensions. For example, weakened connectivity of the dorsal cognitive control network is thought to underlie cognitive deficits (Helm et al., 2018; Li et al., 2018). Cognitive function often depends on the integrity of large‐scale structural white matter connections in the underlying brain networks (Petersen & Sporns, 2015). Diffusion tensor imaging (DTI) is a technique to be used to quantify water diffusion in tissue, allowing for the investigation of diffusion in the brain, which is used to gain information about the integrity of white matter (Charlton et al., 2009; Repple et al., 2019). Diffusion anisotropy exists in systems where water diffusion is more impeded in some directions than in others. The simplest way to quantify diffusion anisotropy is through a standard metric called fractional anisotropy (FA). FA is a useful quantity that can be used for cross‐subject comparisons because it is a computable voxel and a scalar value independent of local fiber orientation (and therefore an objective and direct measure). FA is regarded as a quantitative indicator of white matter coherence and has become the most commonly used DTI measure in psychiatric research (Repple et al., 2017). FA is often used to represent the integrity index of white matter fibers (Smith et al., 2007). At present, many studies have shown that FA is reduced in patients with depression (Ganzola et al., 2018; Li et al., 2020). However, it remains unclear whether there is a link between decreased reasoning and problem‐solving and white matter integrity in patients with depression. Thus, the purpose of this study was to explore the relationship between the ability of reasoning and problem‐solving and white matter integrity in patients with depression.

## 2 MATERIALS AND METHODS

### 2.1 Participants

All studies were approved by the Ethics Committee of Zhumadian Second People's Hospital. Participants were recruited through the outpatient and inpatient departments of Zhumadian Second People's Hospital and gave written informed consent. All examinations of the subjects were completed on the same day, and if there was a conflict with the treatment schedule, the neuropsychological tests and MRI were usually performed in no more than 2 days.

Thirty‐three MDD participants who met DSM‐IV criteria for MDD and 24 healthy volunteers (HVs) were included. Inclusion criteria for MDD participants included: (1) 18−50 years of age; (2) meet the U.S. Diagnostic and Statistical Manual (Fourth Edition, DSM‐IV) diagnosis of MDD; (3) Hamilton Depression Rating Scale‐17 item score ≥ 17; (4) the number of depressive episodes in patients was more than two times, and there was no regular use of psychotropic drugs in the past 2 months; (5) Han nationality; and (6) right‐handed. Exclusion criteria included: (1) combined with other psychiatric disorders such as bipolar disorder or schizophrenia (comorbid anxiety disorders were not excluded); (2) history of cerebral organic diseases, or history of cerebration injury, electrical shock treatment, or other serious physical diseases; (3) alcohol or substance abuse; (4) intellectual disability; (5) pregnant and lactating women; (6) metal implants or paramagnetic objects within the body which may pose a risk to the subject or interfere with the MRI scan; and (7) claustrophobia.

### 2.2 Clinical and neuropsychological measures

The Hamilton Depression Rating Scale, the Hamilton Anxiety Rating Scale, and the Young Mania Rating Scale assessed clinician‐rated depression, anxiety, and mania severity, respectively. The higher the scores on these questionnaires, the more severe the symptomatology is.

### 2.3 Reasoning and problem‐solving tests

Neuropsychological assessment battery (NAB) maze tests were used to assess the ability of reasoning and problem‐solving (Rodriguez‐Jimenez et al., 2012). All participants were instructed to complete the test under the guidance of an experienced physician. The test asked participants to draw a path from an entrance to an exit in a maze. The lines drawn must not intersect with the original segments of the maze, and they should not cut corners. The experimenter was timed after announcing the “start.” In the test, subjects can use their fingers or imagination to find their way out of the maze before starting to draw lines.

NAB mazes of A, B, and C have a time limit of 30 s; NAB maze of D has a time limit of 120 s; and NAB mazes of E, F, and G have a time limit of 240 s. If the subject did not get out of the maze within the specified time, the score would be zero. If the subject got out of the maze within the specified time, the score would be given according to the actual time he or she completed the maze. Add up all the maze scores to get the original score. Three consecutive zeros terminate the test.

### 2.4 Neuroimaging data acquisition and processing

DTI was performed on a GE HCGEHC 3.0‐T scanner (Signa HDxt, GE, USA) at Nuclear Magnetic Laboratory in Zhumadian Second People's Hospital. Using a sponge pad to secure the subject's head and keep him or her awake and quiet. DTI, T1‐ and T2‐weighted MRI images were scanned successively and acquired. T1‐weighted images were collected using a 3D fast spoiled gradient‐echo sequence with the following parameters: Time Repeat (TR)/Time Echo (TE) = 6.8/2.5 ms, flip angle = 7°, field of view = 256 × 256 mm (Ferrari et al., 2010), image matrix = 240 × 240, voxel size = 1 mm (Geraets et al., 2021). DTI was acquired using a single‐short spin‐echo planar imaging sequence with the following parameters: TR = 13,000, field of view  = 128 × 128 mm^2^, *b* value = 0, 1000 s/mm^2^, layer thickness = 3 mm, layer spacing = 0 mm.

Using the PANDA software (http://www.nitrc.org/projects/panda/), pretreatment and analysis of the DTI data were done. The pretreatment of DTI data includes format conversion, eddy current, head movement correction, and scalp removal. After the FA graph was calculated, tract‐based spatial statistics was used for voxel statistical analysis of FA data. The FA data are registered into a common FMRIB58FA template (http://cmrm.med.jhmi.edu) located in the MNI152 standard space using the nonlinear registration algorithm FNIRT. Next, an average FA image was created from the continuous scan images of all subjects in this public space and refined to generate an average FA white matter skeleton representing the centers of all fiber bundles common to the whole scanning group with a threshold greater than 0.2. Then, the aligned FA image is projected onto the skeleton by filling it with FA values from the center of the nearest correlation beam. The white matter fiber tracts were studied using a white matter imaging atlas template obtained from the JHU White‐Matter Tractography Atlas, mapped to a standard MNI152 space, and resampled to a resolution of 1 mm.

### 2.5 Data analysis

The demographic and clinical characteristics were compared between MDD groups and HV groups using a *t*‐test or chi‐square test, as appropriate. All analyses were conducted in SPSS 26.0. Like Stone et al. (2016), raw scores were standardized to *t*‐scores. The *t*‐scores were analyzed to examine age, sex, and education effects. The independent sample *t*‐test was used to compare the total NAB maze test scores between the MDD group and the HVs group, with a *p*‐value less than .05 considered statistically significant. Permutation‐based nonparametric inference was used to perform statistical analyses on FA in FSL software, with *n* = 1000 was used to compare the patients and the control group after multiple comparison corrections and threshold‐free cluster enhancement (TFCE), with *p* = .05 as the significant threshold. Analysis of covariance was used to compare the differences in FA between the two groups, with age, gender, and education level as covariates. Pearson correlation analysis was performed with the NAB score, the HAMD score, and course of the disease.

## 3 RESULTS

### 3.1 Demographic variables

The clinical characteristics from the different groups have been summarized (Table 1). There is no difference in sex (*X* ^2^ = 0.922, *p* = .337), age (*F* = 4.577, *p* = .054), and education level (*F* = 0.777, *p* = .066).

**TABLE 1:** Descriptive information.

	MDD	HVs	*t*/*F*/*X* ^2^	*p*
Gender(case, man/women)	15/17	14/10	0.922	.337
Age(year, $\bar{x}$ ± s)	27.09 ± 8.94	32.58 ± 12.10	4.577	.054
Years of education(year, $\bar{x}$ ± s)	11.58 ± 2.51	12.87 ± 2.68	0.777	0.066
HAMD‐17(score, $\bar{x}$ ± s)	20.79 ± 4.03	—	—	—
HAMA(score, $\bar{x}$ ± s)	21.42 ± 10.03	—	—	—
YMRS(score, $\bar{x}$ ± s)	2.03 ± 1.86	—	—	—
Duration (month)	34.85 ± 42.26	—	—	—
The onset of frequency	2.00 ± 2.14	—	—	—

### 3.2 Group differences in ability of reasoning and problem‐solving

**FIGURE 1:**
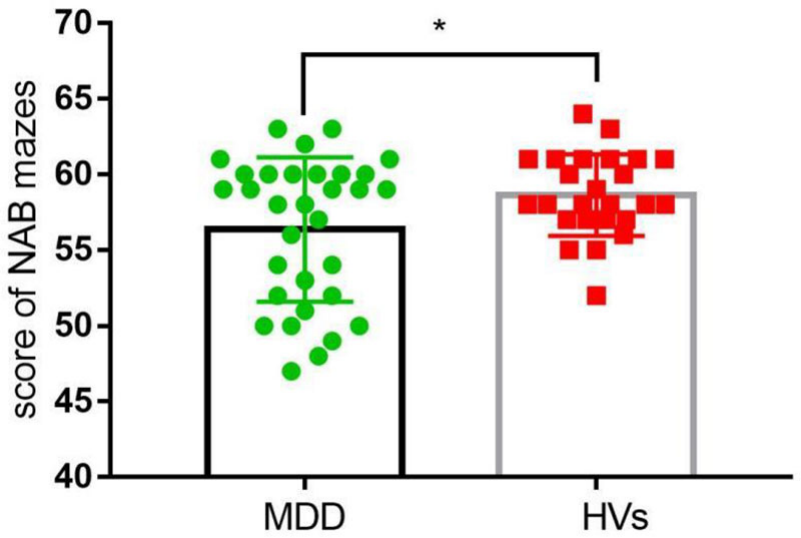
NAB maze tests scores of depressed patients and normal controls. Note: * represents *p* < .05.

In Figure 1, we have calculated the difference in ability of reasoning and problem‐solving between MDD group and HVs group. There is a statistical difference in the total score of NAB mazes (*F* = 11.265, *p* = .037). The HVs group had higher scores than the MDD group.

### 3.3 Group differences in white‐matter tract integrity

**FIGURE 2:**
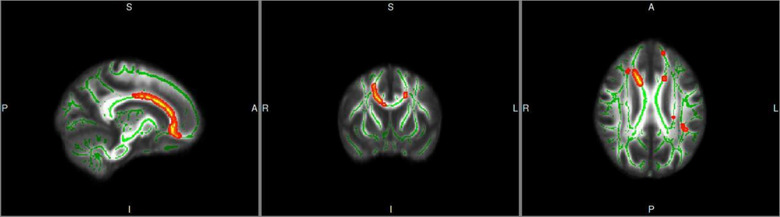
Fiber bundles with reduced FA values in the MDD group compared with the HVs group. Note: The tract‐based spatial statistics skeleton is depicted in green. Significant group differences of white matter integrity are depicted in the red‐yellow color scheme. L, left; R, right; A, anterior; S, superior; I, inferior; P, posterior.

Compared with the HVs group, FA values of the body of corpus callosum and cerebral peduncle right in the MDD group were decreased, and the difference was statistically significant (*p* < .05, TFCE correction). White matter fiber bundles were divided according to JHU‐ICBM‐DTI‐81 Atlas (see Table 2 and Figure 2).

**TABLE 2:** Fractional anisotropy in white matter shows significant differences between the HVs group and the MDD group.

White matter	MNI coordinates (mm)			Cluster Size	*p*
	*X*	*Y*	*Z*		
Body of corpus callosum	−17	4	31	2929	.012
Cerebral peduncle right	30	−42	33	1334	.024

*Note*. Montreal Neurological Institute (MNI) is the brain coordinates of the Montreal Neuroscience Institute; FA is the anisotropic fraction.

### 3.4 Correlations between integrity of white matter and dimensional depression variables

**FIGURE 3:**
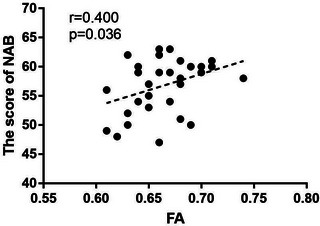
Correlations between white matter integrity and ability of reasoning and problem‐solving.

As shown in Table 3, there was no significant correlation between FA of the body of corpus callosum, cerebral peduncle right, and NAB maze scoring in the HVs group. FA of the body of corpus callosum in depressed patients was significantly correlated with the NAB maze score (*r* = 0.400, *p* = .036) (Figure 3). There was no correlation with HAMD score (*r* = 0.065, *p* = .723), duration of disease (*r* = −0.106, *p* = .558), and onset of frequency (*r* = −0.152, *p* = .400). FA of right anterior corona radiata in depressed patients was not correlated with NAB maze score (*r* = 0.150, *p* = .405), HAMD score (*r* = 0.005, *p* = .980), duration of disease (*r* = 0.138, *p* = .443), and onset of frequency (*r* = 0.166, *p* = .356).

**TABLE 3:** Correlations between integrity of white matter and dimensional depression variables

	The score of NAB mazes		HAMD		Duration		Onset of frequency	
	*R*	*p*	*r*	*p*	*r*	*P*	*r*	*p*
Body of corpus callosum (FA)	0.400	.036	0.065	.723	−0.106	.558	−0.152	.400
Right anterior corona radiata	0.150	.405	0.005	.980	0.138	.443	0.166	.356

## 4 DISCUSSION

In this study, we found that compared with the HVS group, the MDD group had poor ability of reasoning and problem‐solving. This corroborates previous studies about a relation between depression and cognitive deficits, showing that cognitive impairment represents a core feature of depression and manifests itself in multiple domains (Poletti et al., 2017; Rock et al., 2014). In addition, consistent with other studies (Chen et al., 2021; Ji et al., 2022), we found a lower FA value in the MDD group compared to the HVs group. It is mainly found in the body of corpus callosum and right cerebral peduncle.

Second, we found fiber integrity of the body of the corpus callosum was positively correlated with ability of reasoning and problem‐solving. The corpus callosum is composed of more than 100 million axons crisscrossing each other between the two cerebral hemispheres, forming the largest interhemispheric white matter network in the human brain (Rajan et al., 2020). At the same time, it forms an important part of brain function, supporting higher‐order processing throughout the cortex, from the prefrontal system to the visual system (Cyprien et al., 2016; Won et al., 2016). Ballmaier et al. (2008) found callosal thinning in both the genu and splenium, which correlated with memory and attention functioning, but only in the late‐onset MDD group. Given the functional anatomy of the corpus callosum, it plays a pivotal role in the integration of interhemispheric information and higher cognitive functions. Thus, regional structural changes in the corpus callosum might contribute to the impaired cognition and emotional regulation. At present, more and more studies have reported the relationship between corpus callosum changes and affective disorders (Lee et al., 2020; Lin et al., 2020). Studies have found that corpus callosum damage or thinning has been shown to be associated with decreased function in executive function, attention, working memory, processing speed, verbal fluency, and memory tests in both healthy and patient groups (Redmond et al., 2018; Van Schependom et al., 2017). Our study found that the integrity of white matter fibers of the body of corpus callosum correlates with reasoning and problem‐solving. This may be related to the anatomy of the body of the corpus callosum. Decreased integrity of white matter fibers of the body of corpus callosum leads to a decline in reasoning and problem‐solving ability, which in turn leads to a decline in cognitive function in patients with depression. Previous studies have found that maturation in the corpus callosum corresponds to cognitive processes during childhood and adolescence (Cowell et al., 1992; Giedd et al., 1996). Developmental impairment or altered trajectory may contribute to the development of the MDD. Therefore, whether the corpus callosum defects are caused by developmental disorders that lead to cognitive dysfunction needs to be further investigated.

Conversely, studies have found no significant difference in integrity of white matter fiber between MDD and HVs (Chang et al., 2018). This may relate with the age difference between the subjects. Although the MDD matched with the HVs in age, and we analyzed age as a covariate, but the interference caused by the large age span still could not be excluded. A few studies have found the integrity of white matter fibers of the corpus callosum is not only unrelated to cognitive function in MDD, but also to depression severity (Emsell et al., 2017). Our study found that the integrity of white matter fibers in the corpus callosum was not associated with HAMD scores, duration, and the onset of frequency. But, there is a significant correlation between the ability of reasoning and the ability to solve problems in MDD. Of course, we speculate that the decreased integrity of fibers in the corpus callosum in patients with depression may be an endophenotype of the decreased reasoning ability to solve problems. In the future, we will further explore whether depressed patients' white matter fiber integrity increases with their increased ability to reason and solve problems.

In addition, depressed patients with lower FA in the right cerebral peduncle than normal subjects, and is not related to reasoning or problem‐solving ability. The cerebral peduncle is the ventral part of the midbrain. It consists of the downstream fibers of the cerebral cortex leading to the cerebellum, medulla, and spinal cord. Currently, studies have found that the cerebral peduncle is associated with limb motor function (Domi et al., 2020). For example, FA in the cerebral peduncles may be used to predict extremity functions for patients with either hemorrhagic or ischemic stroke (Koyama et al., 2018). It has been suggested that patients with a smaller ipsilateral cerebral peduncle tend to better maintain motor function (Mullin et al., 2016). Anatomically, the striatum is the main input nucleus to the basal ganglia and is known for coordinating casual motor programs (Obeso et al., 2002). The study identifies a novel pioneering role for the striatal direct pathway in the proper assembly of ascending and descending axon bundles within the internal capsule and cerebral peduncle (Ehrman et al., 2022). This further suggests that the cerebral peduncle is associated with motor function. Moreover, this could explain that the FA of the cerebral peduncle is not related to the ability to reason and solve problems, but the lower FA of the cerebral peduncle in depressed patients than in normal subjects needs to be further explored. We speculate that this could be related to the reduced activity in depressed patients.

In conclusion, the decrease of white matter fiber integrity in the body of the corpus callosum, results in the decrease of coordination ability of the left and right hemispheres in depression patients, which may be the reason for the decrease in the ability of reasoning and problem‐solving.

## 5 LIMITATIONS

This study should be considered with the following limitations. First, the data were cross‐sectional. Therefore, we cannot confirm that imperfection of white matter fibers of the corpus callosum is an endophenotype of cognitive dysfunction in patients with depression. And that is what we plan to do next. Second, the sample size is modest, with 33 MDD patients and 24 HVs. Nevertheless, the integrity of white matter fibers of the corpus callosum was not strongly correlated with cognitive function in depression. Previous studies have found that the genu area of the corpus callosum is smaller in adults with early‐onset depression (Guo et al., 2012; Lyoo et al., 2002). We did not stage the onset of depression in early or late, which may be the reason why the experimental results were not significant. Third, although the patient was followed up for 2 months to see if she had turned manic, the possibility of bipolar disorder could not be completely ruled out. It has been found that the fibrous integrity of the corpus callosum is lower in bipolar disorder than in unipolar depression (Han et al., 2019; Wise et al., 2016). In future studies, we will extend the follow‐up of patients with depression to improve the accuracy of the study.

## AUTHOR CONTRIBUTIONS

Zhiren Wang and Fude Yang developed the concept and design of this study. Ruihua Ma performed the experiments and analyzed the data. Yang Luo, Sijia Liu, Xuan Wang, Hua Guo, Meng Zhao, Nan Chen, Panqi Liu, Jing Shi, Yingna Li, Yunlong Tan, Shuping Tan, and Li Tian restructured, polished, and revised the manuscript. All authors contributed to the article and approved the submitted version.

## CONFLICT OF INTEREST STATEMENT

All authors declare that they have no conflict of interest.

### PEER REVIEW

The peer review history for this article is available at https://publons.com/publon/10.1002/brb3.3047.

## Data Availability

The datasets used and/or analyzed during the current study are available from the corresponding author upon reasonable request.

## References

[brb33047-bib-0001] Ballmaier, M. , Kumar, A. , Elderkin‐Thompson, V. , Narr, K. L. , Luders, E. , Thompson, P. M. , Hojatkashani, C. , Pham, D. , Heinz, A. , & Toga, A. W. (2008). Mapping callosal morphology in early‐ and late‐onset elderly depression: An index of distinct changes in cortical connectivity. Neuropsychopharmacology (New York, N.Y.), 33(7), 1528–1536. 10.1038/sj.npp.1301538 PMC2810852 17712348

[brb33047-bib-0002] Chang, M. , Womer, F. Y. , Edmiston, E. K. , Bai, C. , Zhou, Q. , Jiang, X. , Wei, S. , Wei, Y. , Ye, Y. , Huang, H. , He, Y. , Xu, K. , Tang, Y. , & Wang, F. (2018). Neurobiological commonalities and distinctions among three major psychiatric diagnostic categories: A structural MRI study. Schizophrenia Bulletin, 44(1), 65–74. 10.1093/schbul/sbx028 29036668 PMC5768040

[brb33047-bib-0003] Chapman, C. H. , Zhu, T. , Nazem‐Zadeh, M. , Tao, Y. , Buchtel, H. A. , Tsien, C. I. , Lawrence, T. S. , & Cao, Y. (2016). Diffusion tensor imaging predicts cognitive function change following partial brain radiotherapy for low‐grade and benign tumors. Radiotherapy and Oncology, 120(2), 234–240. 10.1016/j.radonc.2016.06.021 27418525 PMC5003665

[brb33047-bib-0004] Charlton, R. A. , Lamar, M. , Zhang, A. , Yang, S. , Ajilore, O. , & Kumar, A. (2014). White‐matter tract integrity in late‐life depression: Associations with severity and cognition. Psychological Medicine, 44(7), 1427–1437. 10.1017/S0033291713001980 24041297 PMC4310501

[brb33047-bib-0005] Charlton, R. A. , Schiavone, F. , Barrick, T. R. , Morris, R. G. , & Markus, H. S. (2009). Diffusion tensor imaging detects age‐related white matter change over a two‐year follow‐up which is associated with working memory decline. Journal of Neurology, Neurosurgery and Psychiatry, 81(1), 13–19. 10.1136/jnnp.2008.167288 19710050

[brb33047-bib-0006] Chen, T. , Chen, Z. , & Gong, Q. (2021). White matter‐based structural brain network of major depression. Advances in Experimental Medicine and Biology, 1305, 35–55. 10.1007/978-981-33-6044-0_3 33834393

[brb33047-bib-0007] Cowell, P. E. , Allen, L. S. , Zalatimo, N. S. , & Denenberg, V. H. (1992). A developmental study of sex and age interactions in the human corpus callosum. Developmental Brain Research, 66(2), 187–192. 10.1016/0165-3806(92)90079-C 1606684

[brb33047-bib-0008] Cyprien, F. , De Champfleur, N. M. , Deverdun, J. , Olié, E. , Le Bars, E. , Bonafé, A. , Mura, T. , Jollant, F. , Courtet, P. , & Artero, S. (2016). Corpus callosum integrity is affected by mood disorders and also by the suicide attempt history: A diffusion tensor imaging study. Journal of Affective Disorders, 206, 115–124. 10.1016/j.jad.2016.07.026 27472413

[brb33047-bib-0009] Daly, E. J. , Trivedi, M. H. , Janik, A. , Li, H. , Zhang, Y. , Li, X. , Lane, R. , Lim, P. , Duca, A. R. , Hough, D. , Thase, M. E. , Zajecka, J. , Winokur, A. , Divacka, I. , Fagiolini, A. , Cubala, W. J. , Bitter, I. , Blier, P. , Shelton, R. C. , … Singh, J. B. (2019). Efficacy of esketamine nasal spray plus oral antidepressant treatment for relapse prevention in patients with treatment‐resistant depression: A randomized clinical trial. JAMA Psychiatry, 76(9), 893–903. 10.1001/jamapsychiatry.2019.1189 31166571 PMC6551577

[brb33047-bib-0010] Domi, T. , Deveber, G. , Mikulis, D. , & Kassner, A. (2020). Wallerian degeneration of the cerebral peduncle and association with motor outcome in childhood stroke. Pediatric Neurology, 102, 67–73. 10.1016/j.pediatrneurol.2019.07.004 31607421

[brb33047-bib-0011] Duric, P. , Harhaji, S. , O'may, F. , Boderscova, L. , Chihai, J. , Como, A. , Hranov, G. L. , Mihai, A. , & Sotiri, E. (2018). General practitioners' views towards diagnosing and treating depression in five southeastern European countries. Early Intervention in Psychiatry, 13(5), 1155–1164. 10.1111/eip.12747 30277313 PMC6445789

[brb33047-bib-0012] Ehrman, J. M. , Merchan‐Sala, P. , Ehrman, L. A. , Chen, B. , Lim, H.‐W. , Waclaw, R. R. , & Campbell, K. (2022). Formation of the mouse internal capsule and cerebral peduncle: A pioneering role for striatonigral axons as revealed in *ISl1* conditional mutants. Journal of Neuroscience, 42(16), 3344–3364. 10.1523/JNEUROSCI.2291-21.2022 35273083 PMC9034787

[brb33047-bib-0013] Emsell, L. , Adamson, C. , De Winter, F.‐L. , Billiet, T. , Christiaens, D. , Bouckaert, F. , Adamczuk, K. , Vandenberghe, R. , Seal, M. L. , Sienaert, P. , Sunaert, S. , & Vandenbulcke, M. (2017). Corpus callosum macro and microstructure in late‐life depression. Journal of Affective Disorders, 222, 63–70. 10.1016/j.jad.2017.06.063 28672181

[brb33047-bib-0014] Evans, V. C. , Iverson, G. L. , Yatham, L. N. , & Lam, R. W. (2014). The relationship between neurocognitive and psychosocial functioning in major depressive disorder: A systematic review. Journal of Clinical Psychiatry, 75(12), 1359–1370. 10.4088/JCP.13r08939 25551235

[brb33047-bib-0015] Ferrari, A. J. , Charlson, F. J. , Norman, R. E. , Flaxman, A. D. , Patten, S. B. , Vos, T. , & Whiteford, H. A. (2013). The epidemiological modelling of major depressive disorder: Application for the global burden of disease study 2010. PLoS ONE, 8(7), e69637. 10.1371/journal.pone.0069637 23922765 PMC3726670

[brb33047-bib-0016] Ganzola, R. , Mcintosh, A. M. , Nickson, T. , Sprooten, E. , Bastin, M. E. , Giles, S. , Macdonald, A. , Sussmann, J. , Duchesne, S. , & Whalley, H. C. (2018). Diffusion tensor imaging correlates of early markers of depression in youth at high‐familial risk for bipolar disorder. Journal of Child Psychology and Psychiatry, 59(8), 917–927. 10.1111/jcpp.12879 29488219

[brb33047-bib-0017] Geraets, A. , Schram, M. T. , Jansen, J. , Koster, A. , Dagnelie, P. C. , van Greevenbroek, M. M. J. , Stehouwer, C. D. A. , Verhey, F. R. J. , & Köhler, S. (2021). The relation of depression with structural brain abnormalities and cognitive functioning: The Maastricht study. Psychological Medicine, 52(15), 1–10. 10.1017/S0033291721000222 PMC9772903 33634767

[brb33047-bib-0018] Giedd, J. N. , Rumsey, J. M. , Castellanos, F. X. , Rajapakse, J. C. , Kaysen, D. , Catherine Vaituzis, A. , Vauss, Y. C. , Hamburger, S. D. , & Rapoport, J. L. (1996). A quantitative MRI study of the corpus callosum in children and adolescents. Developmental Brain Research, 91(2), 274–280. 10.1016/0165-3806(95)00193-X 8852379

[brb33047-bib-0019] Goddard, L. , Dritschel, B. , & Burton, A. (1996). Role of autobiographical memory in social problem solving and depression (1996). Journal of Abnormal Psychology, 105(4), 609–616. 10.1037/0021-843X.105.4.609 8952194

[brb33047-bib-0020] Guo, W.‐B. , Liu, F. , Xue, Z.‐M. , Gao, K. , Wu, R.‐R. , Ma, C.‐Q. , Liu, Z.‐N. , Xiao, C.‐Q. , Chen, H.‐F. , & Zhao, J.‐P. (2012). Altered white matter integrity in young adults with first‐episode, treatment‐naive, and treatment‐responsive depression. Neuroscience Letters, 522(2), 139–144. 10.1016/j.neulet.2012.06.027 22721700

[brb33047-bib-0021] Hammar, Å. (2009). Cognitive functioning in major depression: A summary. Frontiers in Human Neuroscience, 3, 26. 10.3389/neuro.09.026.2009 19826496 PMC2759342

[brb33047-bib-0022] Han, K. M. , De Berardis, D. , Fornaro, M. , & Kim, Y. K. (2019). Differentiating between bipolar and unipolar depression in functional and structural MRI studies. Progress in Neuro‐psychopharmacology & Biological Psychiatry, 91, 20–27. 29601896 10.1016/j.pnpbp.2018.03.022

[brb33047-bib-0023] Helm, K. , Viol, K. , Weiger, T. M. , Tass, P. A. , Grefkes, C. , Del Monte, D. , & Schiepek, G. (2018). Neuronal connectivity in major depressive disorder: A systematic review. Neuropsychiatric Disease and Treatment, 14, 2715–2737. 10.2147/NDT.S170989 30425491 PMC6200438

[brb33047-bib-0024] Ji, L. , Jiang, W. , Liu, D. , & Hou, K. (2022). Effect of SIRT1 on white matter neural network in adolescent patients with depression. Frontiers in Psychiatry, 13, 966315. 10.3389/fpsyt.2022.966315 36177213 PMC9513552

[brb33047-bib-0025] Koyama, T. , Koumo, M. , Uchiyama, Y. , & Domen, K. (2018). Utility of fractional anisotropy in cerebral peduncle for stroke outcome prediction: Comparison of hemorrhagic and ischemic strokes. Journal of Stroke & Cerebrovascular Diseases, 27(4), 878–885. 10.1016/j.jstrokecerebrovasdis.2017.10.022 29174878

[brb33047-bib-0026] Lee, S. , Pyun, S.‐B. , Choi, K. W. , & Tae, W.‐S. (2020). Shape and volumetric differences in the corpus callosum between patients with major depressive disorder and healthy controls. Psychiatry Investigation, 17(9), 941–950. 10.30773/pi.2020.0157 32933236 PMC7538242

[brb33047-bib-0027] Lemoult, J. , & Gotlib, I. H. (2019). Depression: A cognitive perspective. Clinical Psychology Review, 69, 51–66. 10.1016/j.cpr.2018.06.008 29961601 PMC11884012

[brb33047-bib-0028] Li, B. J. , Friston, K. , Mody, M. , Wang, H. N. , Lu, H. B. , & Hu, D. W. (2018). A brain network model for depression: From symptom understanding to disease intervention. CNS Neuroscience & Therapeutics, 24(11), 1004–1019. 29931740 10.1111/cns.12998 PMC6490158

[brb33047-bib-0029] Li, Z. , Liu, W. , Xiao, C. , Wang, X. , Zhang, X. , Yu, M. , Hu, X. , & Qian, L. (2020). Abnormal white matter microstructures in Parkinson's disease and comorbid depression: A whole‐brain diffusion tensor imaging study. Neuroscience Letters, 735, 135238. 10.1016/j.neulet.2020.135238 32645398

[brb33047-bib-0030] Lin, C. , Huang, C.‐M. , Fan, Y.‐T. , Liu, H.‐L. , Chen, Y.‐L. , Aizenstein, H. J. , Lee, T. M.‐C. , & Lee, S.‐H. (2020). Cognitive reserve moderates effects of white matter hyperintensity on depressive symptoms and cognitive function in late‐life depression. Frontiers in Psychiatry, 11, 249. 10.3389/fpsyt.2020.00249 32322221 PMC7158948

[brb33047-bib-0031] Lyoo, I. K. , Kwon, J. S. , Lee, S. J. , Han, M. H. , Chang, C.‐G. , Seo, C. S. , Lee, S. I. K. , & Renshaw, P. F. (2002). Decrease in genu of the corpus callosum in medication‐naïve, early‐onset dysthymia and depressive personality disorder. Biological Psychiatry, 52(12), 1134–1143. 10.1016/S0006-3223(02)01436-1 12488058

[brb33047-bib-0032] Miskowiak, K. W. , Kjærstad, H. L. , Meluken, I. , Petersen, J. Z. , Maciel, B. R. , Köhler, C. A. , Vinberg, M. , Kessing, L. V. , & Carvalho, A. F. (2017). The search for neuroimaging and cognitive endophenotypes: A critical systematic review of studies involving unaffected first‐degree relatives of individuals with bipolar disorder. Neuroscience and Biobehavioral Reviews, 73, 1–22. 10.1016/j.neubiorev.2016.12.011 27979650

[brb33047-bib-0033] Mullin, J. P. , Soni, P. , Lee, S. , Jehi, L. , Naduvil Valappi, A. M. , Bingaman, W. , & Gonzalez‐Martinez, J. (2016). Volumetric analysis of cerebral peduncles and cerebellar hemispheres for predicting hemiparesis after hemispherectomy. Neurosurgery, 79(3), 499–507. 10.1227/NEU.0000000000001307 27322806

[brb33047-bib-0034] Nolen‐Hoeksema, S. (1991). Responses to depression and their effects on the duration of depressive episodes. Journal of Abnormal Psychology, 100(4), 569–582. 10.1037/0021-843X.100.4.569 1757671

[brb33047-bib-0035] Noreen, S. , & Dritschel, B. (2022). In the here and now: Future thinking and social problem‐solving in depression. PLoS ONE, 17(6), e0270661. 10.1371/journal.pone.0270661 35771846 PMC9246169

[brb33047-bib-0036] Obeso, J. A. , Rodriguez‐Oroz, M. C. , Rodriguez, M. , Arbizu, J. , & Giménez‐Amaya, J. M. (2002). The basal ganglia and disorders of movement: pathophysiological mechanisms. News in Physiological Sciences, 17(2), 51–55. 11909992 10.1152/nips.01363.2001

[brb33047-bib-0037] Petersen, S. E. , & Sporns, O. (2015). Brain networks and cognitive architectures. Neuron (Cambridge, Mass.), 88(1), 207–219. 10.1016/j.neuron.2015.09.027 PMC4598639 26447582

[brb33047-bib-0038] Poletti, S. , Aggio, V. , Brioschi, S. , Dallaspezia, S. , Colombo, C. , & Benedetti, F. (2017). Multidimensional cognitive impairment in unipolar and bipolar depression and the moderator effect of adverse childhood experiences: Early stress and cognition in psychiatry. Psychiatry and Clinical Neurosciences, 71(5), 309–317. 10.1111/pcn.12497 28004481

[brb33047-bib-0039] Price, R. B. , & Duman, R. (2020). Neuroplasticity in cognitive and psychological mechanisms of depression: An integrative model. Molecular Psychiatry, 25(3), 530–543. 10.1038/s41380-019-0615-x 31801966 PMC7047599

[brb33047-bib-0040] Priester, M. J. , & Clum, G. A. (1993). Perceived problem‐solving ability as a predictor of depression, hopelessness, and suicide ideation in a college population. Journal of Counseling Psychology, 40(1), 79–85. 10.1037/0022-0167.40.1.79

[brb33047-bib-0041] Rajan, S. , Brettschneider, J. , & Collingwood, J. F. (2020). Regional segmentation strategy for DTI analysis of human corpus callosum indicates motor function deficit in mild cognitive impairment. Journal of Neuroscience Methods, 345, 108870. 10.1016/j.jneumeth.2020.108870 32687851

[brb33047-bib-0042] Redmond, K. J. , Hildreth, M. , Sair, H. I. , Terezakis, S. , Mcnutt, T. , Kleinberg, L. , Cohen, K. J. , Wharam, M. , Horska, A. , & Mahone, E. M (2018). Association of neuronal injury in the genu and body of corpus callosum after cranial irradiation in children with impaired cognitive control: A prospective study. International Journal of Radiation and Oncology in Biology and Physics, 101(5), 1234–1242. 10.1016/j.ijrobp.2018.04.037 PMC6050077 29908790

[brb33047-bib-0043] Repple, J. , Meinert, S. , Grotegerd, D. , Kugel, H. , Redlich, R. , Dohm, K. , Zaremba, D. , Opel, N. , Buerger, C. , Förster, K. , Nick, T. , Arolt, V. , Heindel, W. , Deppe, M. , & Dannlowski, U. (2017). A voxel‐based diffusion tensor imaging study in unipolar and bipolar depression. Bipolar Disorders, 19(1), 23–31. 10.1111/bdi.12465 28239946

[brb33047-bib-0044] Repple, J. , Zaremba, D. , Meinert, S. , Grotegerd, D. , Redlich, R. , Förster, K. , Dohm, K. , Opel, N. , Hahn, T. , Enneking, V. , Leehr, E. J. , Böhnlein, J. , Dzvonyar, F. , Sindermann, L. , Winter, N. , Goltermann, J. , Kugel, H. , Bauer, J. , Heindel, W. , … Dannlowski, U. (2019). Time heals all wounds? A 2‐year longitudinal diffusion tensor imaging study in major depressive disorder. Journal of Psychiatry & Neuroscience, 44(6), 407–413. 31094489 10.1503/jpn.180243 PMC6821510

[brb33047-bib-0045] Rock, P. L. , Roiser, J. P. , Riedel, W. J. , & Blackwell, A. D. (2014). Cognitive impairment in depression: A systematic review and meta‐analysis. Psychological Medicine, 44(10), 2029–2040. 10.1017/S0033291713002535 24168753

[brb33047-bib-0046] Rodriguez‐Jimenez, R. , Bagney, A. , Garcia‐Navarro, C. , Aparicio, A. I. , Lopez‐Anton, R. , Moreno‐Ortega, M. , Jimenez‐Arriero, M. A. , Santos, J. L. , Lobo, A. , Kern, R. S. , Green, M. F. , Nuechterlein, K. H. , & Palomo, T. (2012). The MATRICS consensus cognitive battery (MCCB): Co‐norming and standardization in Spain. Schizophrenia Research, 134(2), 279–284. 10.1016/j.schres.2011.11.026 22192501

[brb33047-bib-0047] Ruan, Q.‐N. , Chen, C. , Jiang, D.‐G. , Yan, W.‐J. , & Lin, Z. (2022). A network analysis of social problem‐solving and anxiety/depression in adolescents. Frontiers in Psychiatry, 13, 921781. 10.3389/fpsyt.2022.921781 36032238 PMC9401098

[brb33047-bib-0048] Santos, J. L. , Aparicio, A. , Bagney, A. , Sánchez‐Morla, E. M. , Rodríguez‐Jiménez, R. , Mateo, J. , & Jiménez‐Arriero, M. Á. (2014). A five‐year follow‐up study of neurocognitive functioning in bipolar disorder. Bipolar Disorders, 16(7), 722–731. 10.1111/bdi.12215 24909395

[brb33047-bib-0049] Santos, J. L. , Aparicio, A. , Bagney, A. , Sánchez‐Morla, E. M. , Rodríguez‐Jiménez, R. , Mateo, J. , & Jiménez‐Arriero, M. Á. (2014). A five‐year follow‐up study of neurocognitive functioning in bipolar disorder. Bipolar Disorders, 16(7), 722–731. 10.1111/bdi.12215 24909395

[brb33047-bib-0050] Shao, T. N. , Yin, G. Z. , Yin, X. L. , Wu, J. Q. , Du, X. D. , Zhu, H. L. , Liu, J. H. , Wang, X. Q. , Xu, D. W. , Tang, W. J. , & Hui, L. (2017). Elevated triglyceride levels are associated with cognitive impairments among patients with major depressive disorder. Comprehensive Psychiatry, 75, 103–109. 10.1016/j.comppsych.2017.03.007 28342378

[brb33047-bib-0051] Smith, S. M. , Johansen‐Berg, H. , Jenkinson, M. , Rueckert, D. , Nichols, T. E. , Miller, K. L. , Robson, M. D. , Jones, D. K. , Klein, J. C. , Bartsch, A. J. , & Behrens, T. E. J. (2007). Acquisition and voxelwise analysis of multi‐subject diffusion data with tract‐based spatial statistics. Nature Protocols, 2(3), 499–503. 10.1038/nprot.2007.45 17406613

[brb33047-bib-0052] Stone, W. S. , Mesholam‐Gately, R. I. , Giuliano, A. J. , Woodberry, K. A. , Addington, J. , Bearden, C. E. , Cadenhead, K. S. , Cannon, T. D. , Cornblatt, B. A. , Mathalon, D. H. , Mcglashan, T. H. , Perkins, D. O. , Tsuang, M. T. , Walker, E. F. , Woods, S. W. , Mccarley, R. W. , Heinssen, R. , Green, M. F. , Nuechterlein, K. , & Seidman, L. J. (2016). Healthy adolescent performance on the MATRICS consensus cognitive battery (MCCB): Developmental data from two samples of volunteers. Schizophrenia Research, 172(1‐3), 106–113. 10.1016/j.schres.2016.02.003 26896388 PMC5410891

[brb33047-bib-0053] Van Schependom, J. , Gielen, J. , Laton, J. , Sotiropoulos, G. , Vanbinst, A.‐M. , De Mey, J. , Smeets, D. , & Nagels, G. (2017). The effect of morphological and microstructural integrity of the corpus callosum on cognition, fatigue and depression in mildly disabled MS patients. Magnetic Resonance Imaging, 40, 109–114. 10.1016/j.mri.2017.04.010 28438714

[brb33047-bib-0054] Wise, T. , Radua, J. , Nortje, G. , Cleare, A. J. , Young, A. H. , & Arnone, D. (2016). Voxel‐based meta‐analytical evidence of structural disconnectivity in major depression and bipolar disorder. Biological Psychiatry, 79(4), 293–302. 10.1016/j.biopsych.2015.03.004 25891219

[brb33047-bib-0055] Won, E. , Choi, S. , Kang, J. , Kim, A. , Han, K.‐M. , Chang, H. S. , Tae, W. S. , Son, K. R. , Joe, S.‐H. , Lee, M.‐S. , & Ham, B.‐J. (2016). Association between reduced white matter integrity in the corpus callosum and serotonin transporter gene DNA methylation in medication‐naive patients with major depressive disorder. Translational Psychiatry, 6(8), e866. 10.1038/tp.2016.137 27505229 PMC5022083

[brb33047-bib-0056] Yamada, S. , Takahashi, S. , Malchow, B. , Papazova, I. , Stöcklein, S. , Ertl‐Wagner, B. , Papazov, B. , Kumpf, U. , Wobrock, T. , Keller‐Varady, K. , Hasan, A. , Falkai, P. , Wagner, E. , Raabe, F. J. , & Keeser, D. (2022). Cognitive and functional deficits are associated with white matter abnormalities in two independent cohorts of patients with schizophrenia. European Archives of Psychiatry and Clinical Neuroscience, 272(6), 957–969. 10.1007/s00406-021-01363-8 34935072 PMC9388472

